# REGN-COV2 antibodies prevent and treat SARS-CoV-2 infection in rhesus macaques and hamsters

**DOI:** 10.1126/science.abe2402

**Published:** 2020-10-09

**Authors:** Alina Baum, Dharani Ajithdoss, Richard Copin, Anbo Zhou, Kathryn Lanza, Nicole Negron, Min Ni, Yi Wei, Kusha Mohammadi, Bret Musser, Gurinder S. Atwal, Adelekan Oyejide, Yenny Goez-Gazi, John Dutton, Elizabeth Clemmons, Hilary M. Staples, Carmen Bartley, Benjamin Klaffke, Kendra Alfson, Michal Gazi, Olga Gonzalez, Edward Dick, Ricardo Carrion, Laurent Pessaint, Maciel Porto, Anthony Cook, Renita Brown, Vaneesha Ali, Jack Greenhouse, Tammy Taylor, Hanne Andersen, Mark G. Lewis, Neil Stahl, Andrew J. Murphy, George D. Yancopoulos, Christos A. Kyratsous

**Affiliations:** 1 Regeneron Pharmaceuticals, Inc., Tarrytown, NY 10591, USA.; 2 Southwest National Primate Research Center, Texas Biomedical Research Institute, San Antonio, TX 78245, USA.; 3 BIOQUAL, Rockville, MD 20850, USA.

## Abstract

An urgent global quest for effective therapies to prevent and treat coronavirus disease 2019 (COVID-19) is ongoing. We previously described REGN-COV2, a cocktail of two potent neutralizing antibodies (REGN10987 and REGN10933) that targets nonoverlapping epitopes on the severe acute respiratory syndrome coronavirus 2 (SARS-CoV-2) spike protein. In this report, we evaluate the in vivo efficacy of this antibody cocktail in both rhesus macaques, which may model mild disease, and golden hamsters, which may model more severe disease. We demonstrate that REGN-COV-2 can greatly reduce virus load in the lower and upper airways and decrease virus-induced pathological sequelae when administered prophylactically or therapeutically in rhesus macaques. Similarly, administration in hamsters limits weight loss and decreases lung titers and evidence of pneumonia in the lungs. Our results provide evidence of the therapeutic potential of this antibody cocktail.

## A beneficial cocktail

Since the start of the coronavirus disease 2019 (COVID-19) pandemic, considerable effort has gone into generating and characterizing neutralizing antibodies that could be used as therapeutics. Studies in humanized mice and convalescent humans led to the development of a cocktail of two potent antibodies that simultaneously bind to the severe acute respiratory syndrome coronavirus 2 (SARS-CoV-2) spike protein and prevent the virus from entering host cells. Baum *et al.* evaluated the efficacy of this cocktail, REGN-COV2, in rhesus macaques, which may model mild disease, and in golden hamsters, which present more severe symptoms. The antibody cocktail provided benefits in both models when administered either prophylactically or therapeutically and is currently in clinical trials.

*Science*, this issue p. 1110

## Teaser

In vivo efficacy of the REGN-COV2 antibody cocktail is evaluated in the rhesus macaque and golden hamster models of SARS-CoV-2 infection.

Fully human monoclonal antibodies (mAbs) are a promising class of therapeutics against severe acute respiratory syndrome coronavirus 2 (SARS-CoV-2) infection (*1*). To date, multiple studies have described the discovery and characterization of potent neutralizing mAbs targeting the spike glycoprotein of SARS-CoV-2 (*2*–*11*). However, evaluation of the efficacy of these antibodies in vivo is only beginning to emerge and has largely focused on the prophylactic setting (*6*, *10*, *12*). Furthermore, because the animal models of SARS-CoV-2 infection and coronavirus disease 2019 (COVID-19) are still being developed, no single model has emerged as being more relevant for human disease. Indeed, based on the extremely diverse manifestations of COVID-19 in humans, multiple animal models may be needed to mimic various settings of human infection. The rhesus macaque model is widely used to assess efficacy of therapeutics and vaccines and displays a transient and mild course of the disease (*13*–*20*). On the contrary, the golden hamster model manifests a much more severe form of the disease, accompanied by rapid weight loss and severe lung pathology (*21*–*23*).

We previously described a cocktail of two fully human antibodies, REGN10933 and REGN10987, that bind to spike protein, potently neutralize SARS-CoV-2, and were selected as components of an antiviral antibody cocktail (REGN-COV2) to safeguard against mutational virus escape (*8*, *9*). In this study, we used two different animal models, rhesus macaque and golden hamster, that capture the diverse pathology of SARS-CoV-2 infection and evaluated the in vivo efficacy of this antibody cocktail when used prophylactically or therapeutically. This assessment allows us to compare the performance of the antibodies in diverse disease settings to more comprehensively understand the mechanisms by which mAb therapies may limit viral load and pathology in infected individuals.

**Fig. 1:**
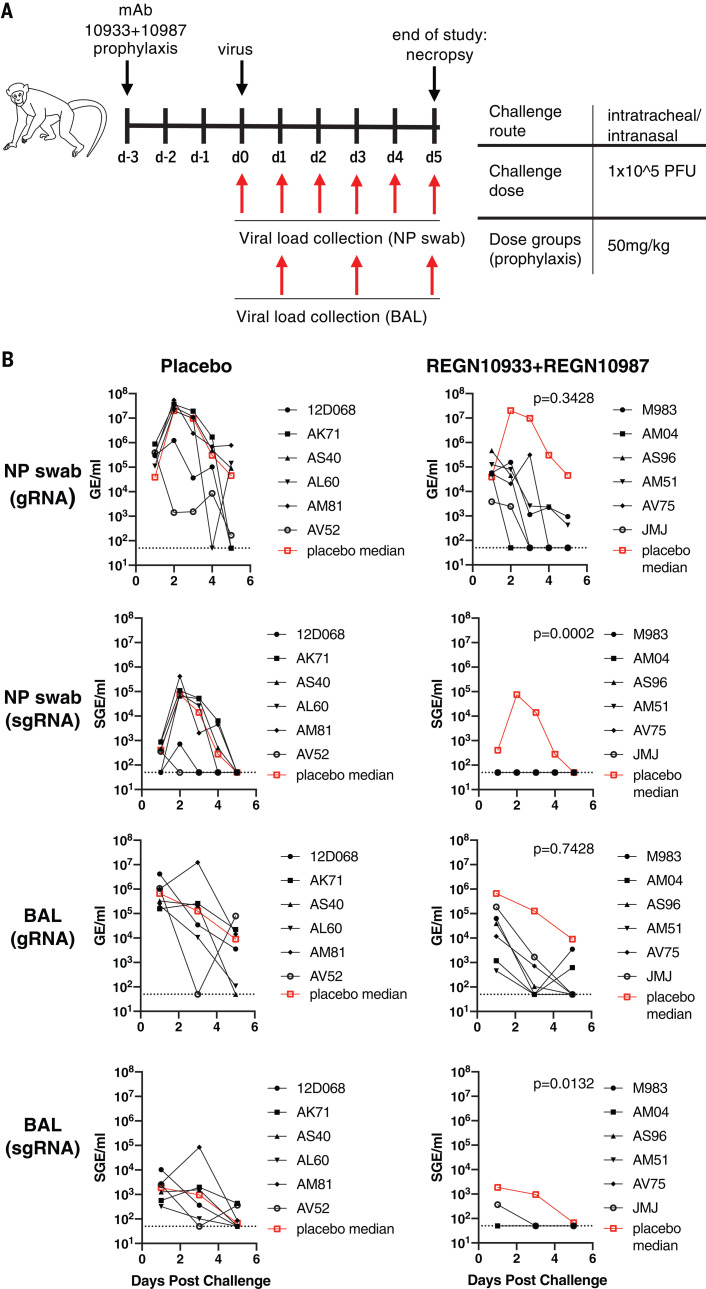
Prophylactic efficacy of REGN-COV2 in the rhesus macaque model of SARS-CoV-2 infection (NHP study 1). (**A**) Overview of study design. d, day. (**B**) Impact of REGN-COV2 prophylaxis on viral gRNA and sgRNA in nasopharyngeal (NP) swabs and BAL fluid. The numbers in the graph legends represent animal codes. The dotted lines indicate limit of detection (LOD = 50 GE/ml for gRNA and LOD = 50 SGE/ml for sgRNA). For detailed statistical analysis, refer to tables S2 and S3. GE, genomic equivalents; SGE, subgenomic equivalents.

To evaluate the ability of REGN-COV2 to protect rhesus macaques from SARS-CoV-2 infection, we initially assessed the impact of antibody administration before virus challenge [nonhuman primate (NHP) study 1]. Six animals were dosed with REGN-COV2 at 50 mg per kilogram of body weight (mg/kg) (25 mg/kg of each antibody) and six with placebo through intravenous administration and challenged with 1 × 10^5^ plaque-forming units (PFU) of virus through intranasal and intratracheal routes 3 days after mAb dosing. Because of the relatively transient nature of the SARS-CoV-2 infection in rhesus macaques, the in-life portion of the study was limited to 5 days. To determine the impact of mAb prophylaxis on viral load in the upper and lower airways, we collected nasopharyngeal swabs on a daily basis and bronchoalveolar lavage (BAL) fluid on days 1, 3, and 5 after challenge (Fig. 1A). Both genomic RNA (gRNA) and subgenomic RNA (sgRNA) (which is made during replication) were measured to assess the impact of mAb prophylaxis on the dynamics of viral replication; whereas gRNA may reflect remaining viral inoculum as well as newly replicating virus, sgRNA should only result from newly replicating virus. For placebo-treated animals, the kinetics of viral load measures was as previously reported, with a peak in viral load on day 2 after challenge, although the majority of animals were still positive for viral RNA in nasal swabs on day 5; even though the kinetics of gRNA and sgRNA were similar, sgRNA levels were about a hundred-fold lower, consistent with what others have reported (*6*, *15*, *16*, *18*). For animals receiving REGN-COV2 prophylaxis, we observed accelerated clearance of gRNA with almost complete ablation of sgRNA in the majority of the animals, showing that REGN-COV2 can almost completely block establishment of virus infection; this pattern was observed across all measurements in both nasopharyngeal swabs and BAL compared with that from placebo animals, demonstrating that mAbs administered prophylactically can greatly reduce viral load in both the upper and lower airways (Fig. 1B).

**Fig. 2:**
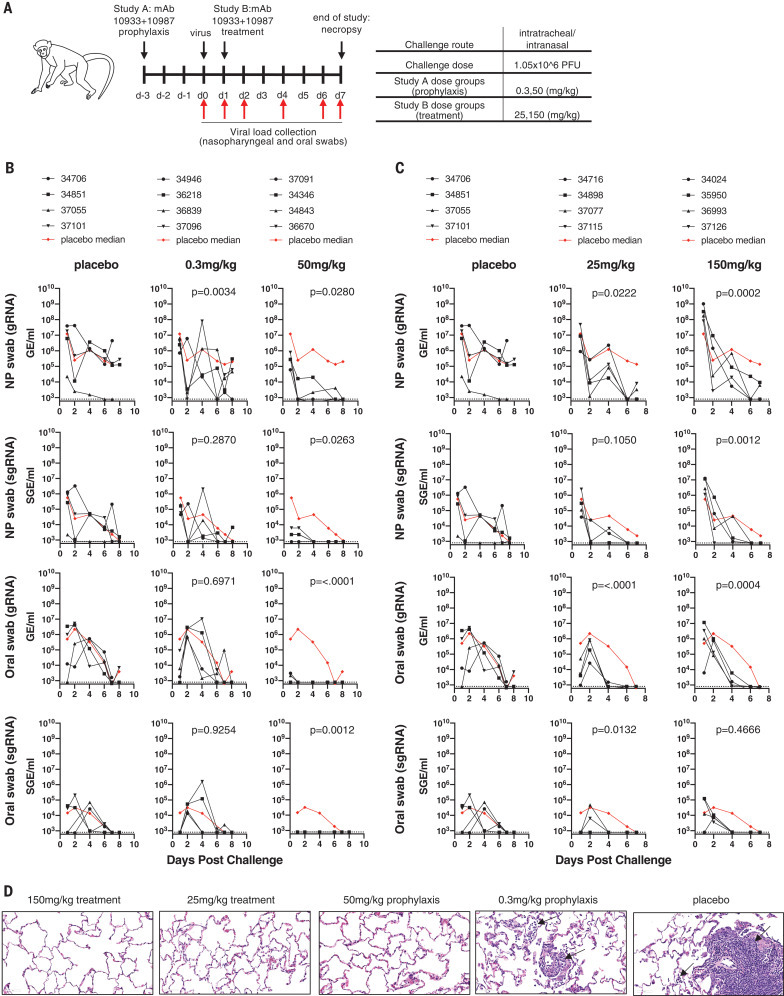
Prophylactic and therapeutic efficacy of REGN-COV2 in the rhesus macaque model of SARS-CoV-2 infection (NHP study 2). (**A**) Overview of study design. (**B**) Impact of REGN-COV2 prophylaxis on viral gRNA and sgRNA in nasopharyngeal swabs and oral swabs [Study A, as shown in (A)]. (**C**) Impact of REGN-COV2 treatment on viral gRNA and sgRNA in nasopharyngeal swabs and oral swabs [Study B, as shown in (A)]. In (B) and (C), the numbers in the graph legends represent animal codes, and the dotted lines indicate limit of detection (LOD = 800 GE/ml for gRNA and LOD = 800 SGE/ml for sgRNA). (**D**) Representative images of histopathology in lungs of treated and placebo animals. The black arrows point to inflammatory cells. For detailed statistical analysis, refer to tables S2 and S3.

A second prophylatic study (NHP study 2) was designed to test whether REGN-COV2 could protect against a 10-fold higher viral inoculum (1.05 × 10^6^ PFU) and compared four animals treated with the 50 mg/kg dose of REGN-COV2 (25 mg/kg of each antibody) with four animals treated with a much lower dose of 0.3 mg/kg and four animals that were administered placebo (Fig. 2A). Nasopharyngeal and oral swabs were collected and used to measure viral gRNA and sgRNA. BAL samples were not collected in this study to minimize the potential impact of the procedure on histopathological analysis of the lung tissue. We observed that 50 mg/kg of REGN-COV2 administered 3 days before virus challenge was once again able to minimize virus replication even when animals were challenged with this 10-fold higher viral inoculum (Fig. 2B), whereas the prophylactic effect was greatly diminished with the 0.3 mg/kg dose. Interestingly, in this study we observed an increased impact of mAb treatment on viral load in oral swabs versus nasopharyngeal swabs, potentially indicating that mAb treatment may affect different physiological sources of virus replication differentially. Additional studies in animal models and humans will be needed to assess this.

Next, we assessed the impact of REGN-COV2 in the treatment setting by dosing four animals challenged with the higher 1 × 10^6^ PFU of SARS-CoV-2 virus at 1 day after infection with 25 or 150 mg/kg of the antibody cocktail (Fig. 2A). By day 1 after challenge, the animals had already reached peak viral load, as measured by both gRNA and sgRNA, mimicking a likely early treatment clinical scenario of COVID-19 disease, because it has been shown that most SARS-CoV-2–infected individuals reach peak viral loads relatively early in the disease course and often before or just at the start of symptom onset (*24*, *25*). Compared with four placebo treated animals, REGN-COV2–treated animals displayed accelerated viral clearance in both nasopharyngeal and oral swab samples, including both gRNA and sgRNA samples (Fig. 2C), clearly demonstrating that the monoclonal antibody cocktail can affect virus load even when administered after infection. Similar to the prophylactic study, the decrease in viral load appeared more dramatic in oral swabs versus nasopharyngeal swabs. Both treatment groups displayed similar kinetics of virus clearance, suggesting that 25 and 150 mg/kg doses demonstrate similar efficacy in this study. The treated animals in the 150 mg/kg group displayed about 10-fold higher titers on day 1, at the time of mAb administration, therefore potentially masking an enhanced effect of a higher drug dose. A similar impact of mAb treatment was observed on gRNA and sgRNA for both nasopharyngeal and oral samples, indicating that the mAb treatment is directly limiting viral replication in these animals (Fig. 2C).

The two antibody components of REGN-COV2 were selected to target nonoverlapping sites on the spike protein to prevent selection of escape mutants, which were readily detectable with a single-mAb treatment (*9*). To assess whether any signs of putative escape mutants are observed in an in vivo setting with authentic SARS-CoV-2 virus, we performed RNA sequencing (RNA-seq) analysis on all RNA samples obtained from all animals from the study. Analysis of the spike protein sequence identified mutations in NHP samples that were not present in the inoculum virus (fig. S1), further indicating that the virus is actively replicating in these animals. However, we did not observe any mutations that were specific to treated animals; all identified mutations were present either in the inoculum or in both treated and placebo animals, indicating that they were likely selected as part of virus replication in NHPs and were not selected by mAb treatment.

We next performed pathology analyses of the lungs of infected animals. All four placebo monkeys showed evidence of lung injury, which was characterized in three monkeys by interstitial pneumonia (Fig. 2D), with minimal to mild infiltration of mononuclear cells (lymphocytes and macrophages) in the septa, perivascular space, and/or pleura. In these three animals, the distribution of lesions was multifocal and involved two to three of the four lung lobes. Accompanying these changes were alveolar infiltration of lymphocytes, increased alveolar macrophages, and syncytial cells. Type II pneumocyte hyperplasia was also observed in occasional alveoli. In the fourth placebo monkey, lung injury was limited to type II pneumocyte hyperplasia, suggestive of a reparative process secondary to type I pneumocyte injury. Overall, the histological lesions observed in the placebo animals were consistent with an acute SARS-CoV-2 infection. In the prophylactic groups, three of four animals in the low-dose (0.3 mg/kg) and one of four animals in the high-dose (50 mg/kg) groups showed evidence of interstitial pneumonia (table S1) that was generally minimal and with fewer histological features when compared with that of the placebo group. In the one affected high-dose group animal, only one of the four lung lobes had a minimal lesion. In the therapeutic treatment groups, two of four low-dose (25 mg/kg) and two of four high-dose (150 mg/kg) treated animals showed evidence of interstitial pneumonia. In all affected low- and high-dose animals, only one of four lung lobes had lesions. Finally, there were no drug-related toxicities observed at any of the doses tested. In summary, the incidence of interstitial pneumonia (the number of animals as well as the number of lung lobes affected) and the severity were reduced in both prophylactic and therapeutic treatment modalities compared with placebo. The analyses demonstrate that prophylactic and therapeutic administration of REGN-COV2 greatly reduced virus-induced pathology in rhesus macaques and showed a clean safety profile.

**Fig. 3:**
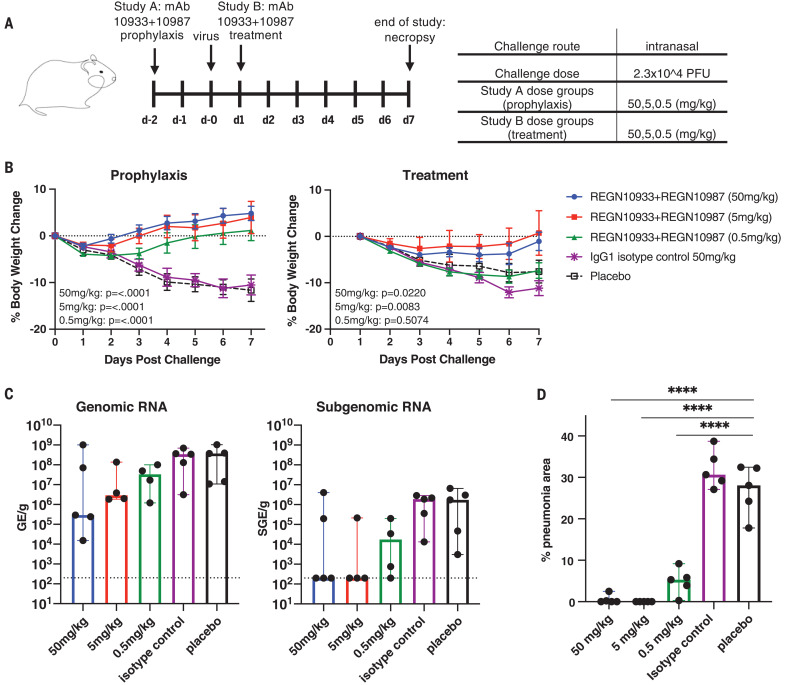
Efficacy of REGN-COV2 in treatment and prophylaxis in the golden Syrian hamster model of SARS-CoV-2 infection. (**A**) Overview of study design. (**B**) Impact of REGN-COV2 on weight loss in prophylaxis and treatment groups. Error bars represent mean with error. IgG1, immunoglobulin G1. (**C**) Impact of REGN-COV-2 prophylaxis on levels of gRNA and sgRNA in hamster lungs (7 days after infection). No statistical significance was observed between any treatment groups and placebo. The dotted lines indicate limit of detection (LOD = 200 GE/ml for gRNA and LOD = 200 SGE/ml for sgRNA), and error bars represent median with 95% confidence intervals. (**D**) Impact of REGN-COV2 prophylaxis on percent area of lung exhibiting pathology typical of pneumonia (****p < 0.0001 indicates significant differences). Error bars represent median with 95% confidence intervals. For detailed statistical analysis, refer to tables S4 and S5.

Unlike rhesus macaques, which present with a mild clinical course of disease and transient virus replication when infected with SARS-CoV-2 that may mimic mild human disease, the golden hamster model is more severe, with animals demonstrating readily observable clinical disease, including rapid weight loss accompanied by very high viral load in the lungs, as well as severe lung pathology. Thus, this model may more closely mimic more severe disease in humans, although more extensive characterization of this model and severe human disease is needed to better understand similarities and differences in pathology. To evaluate the ability of REGN-COV2 to alter the disease course in this model, we designed a study that evaluated the prophylactic and treatment efficacy of the antibodies (Fig. 3A). In the prophylactic study, 25 hamsters were divided into five arms (five animals in each). Administration of 50, 5, or 0.5 mg/kg of REGN-COV2 at 2 days before challenge with a 2.3 × 10^4^ PFU dose of SARS-CoV-2 virus resulted in dramatic protection from weight loss at all doses. This protection was accompanied by decreased viral load in the lungs at the end of the study in the majority of treated animals (day 7 after infection) (Fig. 3C). Evaluation of lung tissue from infected hamsters that were prophylactically treated with placebo or isotype control drug revealed distorted alveoli lined by swollen, hyperplastic type II pneumocytes interspersed with occasional type I single-cell necrosis and alveolar spaces that were filled with large numbers of lymphocytes, macrophages, and neutrophils, occasional syncytial cells, and hemorrhage. These changes were accompanied by variably severe interstitial pneumonia characterized by mixed-cell inflammation (lymphocytes, macrophages, and neutrophils) in the alveolar septa and perivascular spaces accompanied by edema and septal fibrosis. The severity and incidence of alveolar infiltration and interstitial pneumonia were greatly reduced in animals that received REGN-COV2 (Fig. 3D). Compared with placebo- and isotype-treated animals, the percent area of pneumonia in the lungs determined using HALO image analysis software was significantly reduced in all REGN-COV2–treated animals irrespective of doses. Intriguingly, we did observe high gRNA and sgRNA levels in the lungs of a few treated animals, although these individual animals did not show decreased protection from weight loss or more extensive pathology than the animals with much lower viral loads. It is possible that mAb treatment may provide an additional therapeutic benefit in this model that is not directly associated with viral load decrease. Alternatively, it is possible that the increased amounts of detected viral RNA may not necessarily be associated with infectious virus. Because viral replication and lung pathology in the hamster model occur very rapidly, the treatment setting represents a high bar for demonstrating therapeutic efficacy. We used 25 hamsters (five in each of five arms) in a therapeutic study and were able to observe therapeutic benefit in animals treated with 50 and 5 mg/kg doses of REGN-COV2 combination 1 day after viral challenge (Fig. 3B). Taken together, the two hamster studies clearly demonstrate that REGN-COV2 can alter the course of infection in the hamster model of SARS-COV-2 when administered either prophylactically or therapeutically.

In this study, we assessed the in vivo prophylactic and treatment efficacy of the REGN-COV2 mAb cocktail in two animal models, one of mild disease in rhesus macaques and one of severe disease in golden hamsters. Our results demonstrate that the antibodies are efficacious in both animal models, as measured by reduced viral load in the upper and lower airways, by reduced virus-induced pathology in the rhesus macaque model, and by limited weight loss in the hamster model.

The ability of REGN-COV2 to almost completely block detection of subgenomic species of SARS-COV-2 RNA in rhesus macaques matches or exceeds the effects recently shown in vaccine efficacy studies using the same animal models (*18*–*20*, *26*, *27*). Additionally, the observed accelerated reduction of upper-airway virus load in rhesus macaques treated with REGN-COV2 contrasts with the lack of impact on viral load in remdesivir-treated animals, where reduced viral load could only be observed in lower airways with no differences in nasal viral RNA levels (*28*). These findings highlight the therapeutic potential of REGN-COV2 to both protect from and treat SARS-COV-2 disease. Additionally, the impact of REGN-COV2 prophylaxis on viral RNA levels in nasopharyngeal and oral swabs may indicate the potential not only to prevent disease in the exposed individual but also to limit transmission.

Importantly, in our studies, we did not observe any signs of increased viral load or worsening of pathology in the presence of antibodies at either high or low doses in either animal model. Potential for antibody-mediated enhancement of disease is a serious concern for antibody-based therapeutics and vaccines. And although a recent report showed the ability of some anti-spike mAbs to mediate pseudovirus entry into Fcγ receptor–expressing cell lines, these data do not address whether similar behavior would be observed with authentic SARS-CoV-2 virus and primary immune cells (*29*). Our results are consistent with no evidence of enhanced disease in clinical studies that assess convalescent plasma therapy (*30*).

Similarly to most in vivo data generated to date, our in vivo studies were conducted with the D614 spike protein variant of the SARS-CoV-2 virus. A global shift in circulating SARS-CoV-2 to the D614G variant will likely necessitate a transition to use of that variant for in vitro and in vivo studies with SARS-CoV-2 virus in the future (*31*). It is yet not established if pathogenicity and replication dynamics of this variant differ in vivo, and it is equally unclear whether there is an association with severity of human infections (*32*–*34*). Importantly, we have previously demonstrated that the neutralization potency of REGN10933 and REGN10987, as well as the REGN-COV2 combination, was not altered in the presence of this variant, making it likely that the efficacy of the REGN-COV2 combination will extend to the 614G virus (*8*, *35*). Our data provide evidence that REGN-COV2–based therapy may offer clinical benefit in both prevention and treatment settings of COVID-19 disease, where it is currently being evaluated (clinicaltrials.gov NCT04426695, NCT04425629, and NCT04452318).

## Supplementary Materials

science.sciencemag.org/content/370/6520/1110/suppl/DC1 Materials and Methods Figs. S1 and S2 Tables S1 to S5 References (*36*, *37*) MDAR Reproducibility Checklist

## Appendix

View/request a protocol for this paper from *Bio-protocol*.
